# The Interaction Between Microorganisms, Metabolites, and Immune System in the Female Genital Tract Microenvironment

**DOI:** 10.3389/fcimb.2020.609488

**Published:** 2020-12-23

**Authors:** Huanrong Li, Yuqin Zang, Chen Wang, Huiyang Li, Aiping Fan, Cha Han, Fengxia Xue

**Affiliations:** ^1^ Department of Gynecology and Obstetrics, Tianjin Medical University General Hospital, Tianjin, China; ^2^ Department of Gynecology and Obstetrics, Tianjin Key Laboratory of Female Reproductive Health and Eugenic, Tianjin Medical University General Hospital, Tianjin, China

**Keywords:** microenvironment, female genital tract, immunity, metabolites, microbiota

## Abstract

The female reproductive tract microenvironment includes microorganisms, metabolites, and immune components, and the balance of the interactions among them plays an important role in maintaining female reproductive tract homeostasis and health. When any one of the reproductive tract microorganisms, metabolites, or immunity is out of balance, it will affect the other two, leading to the occurrence and development of diseases and the appearance of corresponding symptoms and signs, such as infertility, miscarriage, premature delivery, and gynecological tumors caused by infectious diseases of the reproductive tract. Nutrients in the female reproductive tract provide symbiotic and pathogenic microorganisms with a source of nutrients for their own reproduction and utilization. At the same time, this interaction with the host forms a variety of metabolites. Changes in metabolites in the host reproductive tract are related not only to the interaction between the host and microbiota under dysbiosis but also to changes in host immunity or the environment, all of which will participate in the pathogenesis of diseases and lead to disease-related phenotypes. Microorganisms and their metabolites can also interact with host immunity, activate host immunity, and change the host immune status and are closely related to persistent genital pathogen infections, aggravation of infectious diseases, severe pregnancy outcomes, and even gynecological cancers. Therefore, studying the interaction between microorganisms, metabolites, and immunity in the reproductive tract cannot only reveal the pathogenic mechanisms that lead to inflammation of the reproductive tract, adverse pregnancy outcomes and tumorigenesis but also provide a basis for further research on the diagnosis and treatment of targets.

## Introduction

Different from the high diversity of the gastrointestinal tract, the female genital tract microbiome has low diversity, and it changes dynamically through the female menstrual cycle (Consortium, 2012; Chen et al., 2017). Most microbes have a symbiotic relationship with the host. Accounting for 90–95% of the total bacterial biomass, *Lactobacillus* spp. represents a healthy female genital tract microbiota that produces lactic acid to maintain an acidic microenvironment. It can also inhibit pathogens through competition, adhesion prevention, and the secretion of antibacterial and immunomodulatory substances (Anahtar et al., 2018; Van der Veer et al., 2019). Vaginitis, cervicitis, and pelvic inflammatory disease (PID) will occur if pathogenic bacteria surpass lactobacilli in the female genital tract and can cause uncomfortable symptoms such as increased vulvovaginal discharge, itching, odor, and lower abdominal pain (Workowski and Bolan, 2015). However, there are differences in the microbes between subjects and in the ability of the host to resist dysbiosis that may be related to race, diet, age, living habits, immunity, disease susceptibility, and genetic polymorphism (Consortium, 2012; Anahtar et al., 2018; Chu et al., 2018; Serrano et al., 2019). Furthermore, the dominance of different microflora is not necessarily related to symptoms because partial non-*Lactobacillus*-dominant women do not experience uncomfortable symptoms of vulvovaginitis; hence, we cannot define disease by the number of bacteria alone, and we cannot define dysbiosis without the internal milieu of the host and disease environment (Anahtar et al., 2018; Scott et al., 2019).

**Figure 1:**
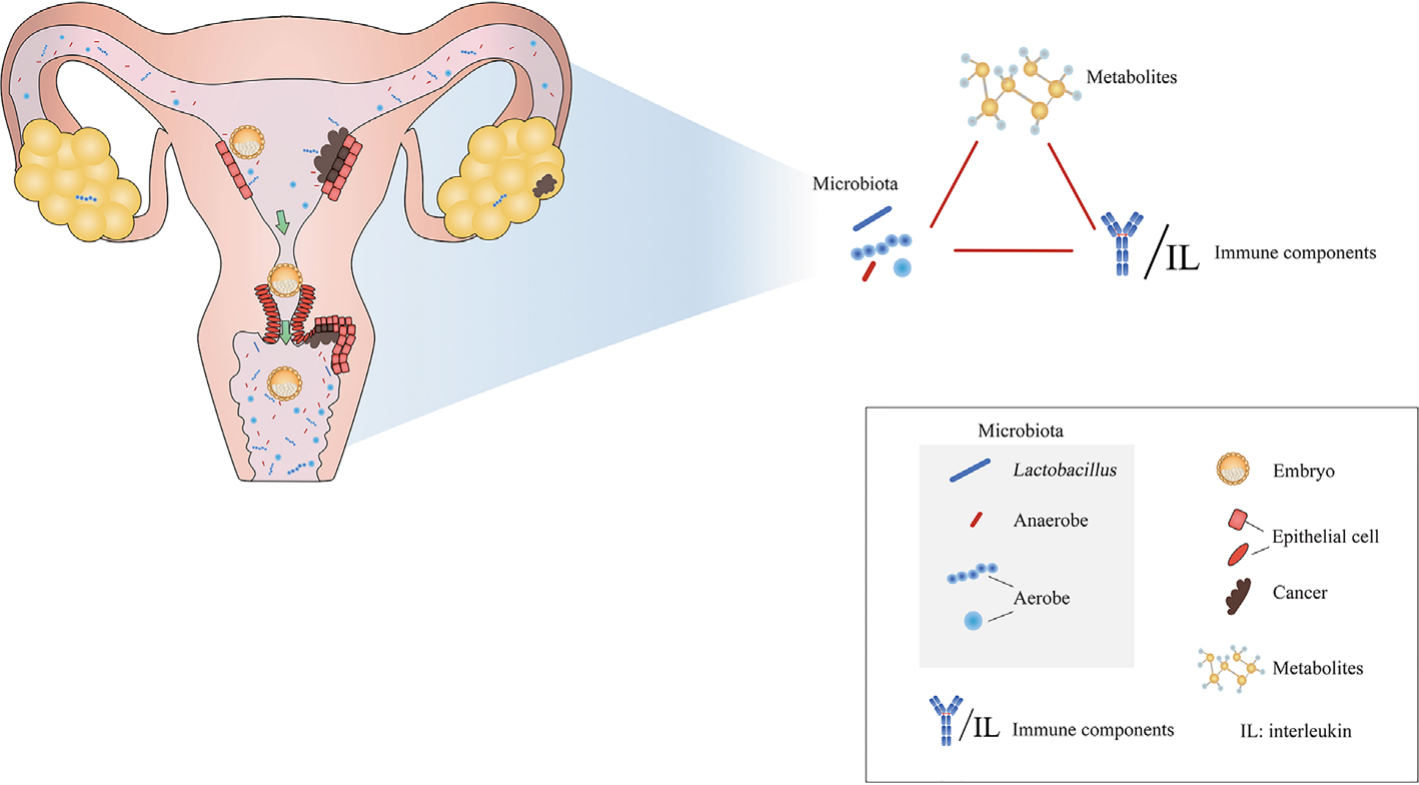
Microenvironmental disorders of the female reproductive tract are closely related to inflammations, adverse pregnancy outcomes, and tumors. Modified from Paweł Łaniewski et al. (2020).

Metabolites in the reproductive tract play an important role in female genital tract inflammation, pregnancy and tumors and can be considered biomarkers of disease severity, diagnosis, and prognosis (Ghartey et al., 2015; McMillan et al., 2015; Ilhan et al., 2019; Song et al., 2019). Metabolites in the female reproductive tract are the substrates, intermediates and byproducts of biochemical reactions caused by the interaction of human nutrients and bacteria, reflecting downstream events of gene expression (Altmäe et al., 2014; McMillan et al., 2015; Turkoglu et al., 2016; Watson and Reid, 2018) (**Figure 1**). These metabolites are better than genome, transcriptome, and proteome substances at predicting the disease phenotype (Altmäe et al., 2014). Genital infections, adverse pregnancy outcomes, and cancers possess different metabolic signatures that are often accompanied by dysbiosis of the female genital tract (Ghartey et al., 2015; Ceccarani et al., 2019; Ilhan et al., 2019). The metabolic pathways affected by these metabolites mainly include amino acids, carbohydrates, and lipid metabolism, which are closely related to life activities (Srinivasan et al., 2015). These activities further affect host cell function, immunity, and disease susceptibility and help maintain the balance of the host’s reproductive tract microenvironment.

The host’s innate and adaptive immune systems perform complex interactions with microorganisms and metabolites (Agostinis et al., 2019; Delgado-Diaz et al., 2019). Microbial ligands bind to host receptors to produce inflammatory factors, chemokines and antimicrobial products to regulate the immune response of the reproductive tract (Hooper et al., 2012).Vaginal dysbiosis cannot only directly cause vaginal epithelial injury through pathogens (Tao et al., 2019), but also indirectly cause vaginal epithelial injury through immune components, which in turn release metabolites into the microenvironment (Olive and Sassetti, 2016; Serrano et al., 2019). This metabolite may be ingested by the vaginal microbiota, leading to increased microbial metabolism, which is beneficial to the growth and reproduction of the microbiota (Serrano et al., 2019). The local competition between the host, pathogen and different immune cells for metabolic precursors will also affect the ability of immune cells to respond effectively to infection, affecting the growth and immunogenicity of the pathogen and further affecting the host response (Hooper et al., 2012; Olive and Sassetti, 2016; Postler and Ghosh, 2017). Therefore, the interaction between microorganisms, metabolites, and immunity in the host reproductive tract microenvironment plays an important role in maintaining the balance of the reproductive tract (Pruski et al., 2018). An imbalance in any part will result in host phenotype changes, disease, and even serious complications. Therefore, this article intended to review the relationship and importance of reproductive tract microorganisms, metabolites, and immunity to obtain a deeper understanding of the reproductive tract microenvironment, reproductive tract diseases and adverse reproductive tract outcomes.

## Normal Vaginal Microenvironment

The vaginal microbiota community state types (CSTs) of women of childbearing age are divided into five categories (Ravel et al., 2011). CST I is dominated by *Lactobacillus crispatus*; CST II by *L. gasseri*; CST V by *L. jensenii*; and CST III by *L. iners*. CST IV belongs to the *Lactobacillus*-deficient type, which is dominated by anaerobic bacteria (classified by bacterial vaginosis, BV), partial aerobic bacteria (classified by aerobic vaginitis, AV) or a modest proportion of *Lactobacillus* spp. (Gajer et al., 2012). The vaginal microbiota is dynamic and occasionally transitions to an intermediate state or a disease state in most normal non-pregnant women. A high Nugent score does not indicate the disease status or microecological disorders (Gajer et al., 2012). The internal milieu of the host will make the microbiota return to a stable state. Factors that cause vaginal microbiota changes are primarily related to menstruation. Others include sexual intercourse, hormonal contraception, antimicrobial agents, use of lubricants, and vaginal douching, but they have less impact than menstrual periods and cause changes in the vaginal microbiota for a shorter duration (Gajer et al., 2012; Mitchell et al., 2012). In addition, in a few women, the vaginal CST does not change with the menstrual cycle and hormonal contraception, but further study by future researchers is needed to determine whether it is related to the metabolic function of bacteria (Gajer et al., 2012; Song et al., 2020).

*Lactobacillus* abundance in the female genital tract is strongly positively correlated with lactate and 4-hydroxyphenylacetate and correlated to a lesser extent with isoleucine, leucine, tryptophan, phenylalanine, aspartate, dimethylamine, sarcosine and pi-methylhistidine, all of which are typically associated with vaginal health (Srinivasan et al., 2015; Ceccarani et al., 2019) (**Table 1**). *L. crispatus* and *L. jensenii* have similar metabolic patterns (Srinivasan et al., 2015), while, *L. crispatus* and *L. iners* have different metabolic characteristics (Pruski et al., 2018). For example, studies have found that the genome of *L. crispatus* is almost twice that of *L. iners* (France et al., 2016). However, the carbon metabolism of *L. iners* is fermented by fewer compounds than *L. crispatus* (Pruski et al., 2018). When the dominant bacteria are *L. crispatus* and/or *L. jensenii*, most of the metabolites in the vagina are amino acids and dipeptide, such as higher levels of ornithine, lysine, glycylproline, phenylalanine (Srinivasan et al., 2015). Similar to BV-related flora, *L. iners* are correlated with amino acid catabolites, such as higher levels of proline, threonine, aspartate, serine, and valinylglutamate (Srinivasan et al., 2015). In addition, *L. iners* often has a symbiotic relationship with *G. vaginalis*, and both produce similar levels of cholesterol-dependent cytolysin (Macklaim et al., 2013). Therefore, the metabolic characteristics of *L. crispatus* and/or *L. jensenii* dominance can be defined as a healthy vaginal microenvironment. However, whether the metabolic characteristics of the non-*Lactobacillus* abundance of some asymptomatic women are similar to those of *L. crispatus* and/or *L. jensenii* dominance needs to be further explored.

**Table 1:** Current existing articles analyzing the correlation between the genital tract flora and metabolites in normal non-pregnant women of reproductive age.

Year	Author	Population	Sample	CST	Metabolites
2019	(Ceccarani et al., 2019)	Healthy women (n = 21), women with BV (n = 20) and women with Chlamydia trachomatis infection (n = 20)	Vaginal swabs	Contains all *Lactobacillus* spp. without distinguishing CST	High levels of lactate, 4-hydroxyphenylacetate, isoleucine, leucine, tryptophan, phenylalanine, aspartate, dimethylamine, sarcosine and pi-methylhistidine
2018	(Parolin et al., 2018)	Healthy women (n = 22), women with Chlamydia trachomatis infection (n = 20), and women with BV (n = 19)	Vaginal swabs	Contains all *Lactobacillus* spp. without distinguishing CST	Higher levels of lactate, 4-hydroxyphenylacetate, diverse amino acids (phenylalanine, glutamate, leucine, threonine, tryptophan, aspartate) and amino acid derivatives(sarcosine)
2015	(Srinivasan et al., 2015)	Women with BV (n = 40) and women without BV (n = 20)	Vaginal fluid	CST-I	1) Higher levels of sugars (maltose, maltotriose, and maltohexose), lipid metabolism biochemicals (such as arachidonate and carnitine), amino acids (ornithine, lysine), dipeptide (glycylproline, phenylalanine) as well as lactate, and urea 2) Lower levels of *N*-acetylneuraminate, succinate, the carnitine precursor deoxycarnitine, the eicosanoid 12-hydroxyeicosatetraenoic acid, the fatty acid 13-hydroxyoctadecadienoic acid, the nucleobase uracil, and glutathione
				CST-III	1) Higher levels of proline, threonine, aspartate, serine, and valinylglutamate 2) Lower levels of glutamate and glycylleucine
				CST-V	Same as CST-I: 1) Higher levels of sugars (maltose, maltotriose, and maltohexose), lipid metabolism biochemicals (such as arachidonate and carnitine), amino acids (ornithine, lysine), dipeptide (glycylproline, phenylalanine) as well as lactate, and urea 2) Lower levels of *N*-acetylneuraminate, succinate, the carnitine precursor deoxycarnitine, the eicosanoid 12-hydroxyeicosatetraenoic acid, the fatty acid 13-hydroxyoctadecadienoic acid, the nucleobase uracil, and glutathione
2015	(McMillan et al., 2015)	Pregnant women (n = 67) and non-pregnant women (n = 64)	Vaginal fluid	CST-I	Higher levels of succinate
2012	(Gajer et al., 2012)	Reproductive age women (n = 32)	Vaginal swabs	CST-III	1) Higher levels of lactate 2) Lower levels of succinate and acetate

CST, Community state types; BV, bacterial vaginosis.

The mucous layer on the surface of female genital tract epithelial cells plays an important role as the first line of defense against microbial invasion (Mirmonsef et al., 2011; Aldunate et al., 2015). When microorganisms break through the line of defense, epithelial cells use pattern recognition receptors (PPRs) to identify microorganisms to produce inflammatory factors and recruit inflammatory cells to resist microbial invasion and colonization. The dominance of *Lactobacillus* in the genital tract is essential, as it inhibits pathogens and maintains immune equilibrium (Smith and Ravel, 2017). Studies have found that the concentration of inflammatory factors in the vagina is very low when *L. crispatus* and *L. jensenii* are dominant (Kyongo et al., 2012). Lactic acid, as a metabolite derived primarily from *Lactobacillus* spp., is also related to reproductive tract immunity (Delgado-Diaz et al., 2019). L-lactic acid produced by *Lactobacillus* spp. can cause an anti-inflammatory response and inhibit the production of proinflammatory cytokines and chemokines induced by toll-like receptor (TLR) in cervical and vaginal epithelial cells at low pH (Delgado-Diaz et al., 2019). In addition, lactic acid can induce the secretion of the anti-inflammatory cytokine interleukin (IL)-10, reduce the production of the proinflammatory cytokine IL-12 in dendritic cells (DCs), and reduce the cytotoxicity of natural killer cells (Ilhan et al., 2019). The anti-inflammatory activity of lactic acid also requires the presence of organic acids produced by microorganisms to maintain vaginal health, mainly by increasing the production of the anti-inflammatory cytokine IL-1RA, inhibiting the proinflammatory signal of the IL-1 cytokine, and slightly reducing the production of the proinflammatory cytokines IL-6 and macrophage inflammatory protein 3 alpha (MIP-3α) (Delgado-Diaz et al., 2019). Therefore, the interaction between the flora, metabolites, and immunity in the healthy reproductive tract is very important for maintaining the health of the reproductive tract. When any one party is imbalanced, it will affect the balance of the reproductive tract.

## Common Reproductive Tract Infections

Female genital tract infections mainly include vaginitis, cervicitis, and PID (Sherrard et al., 2018). The main cause is exogenous pathogen interference or endogenous dysbiosis (Workowski and Bolan, 2015; Song et al., 2020). At present, the relevant research on infectious diseases caused by the interaction between reproductive tract microorganisms, metabolites and the host is mainly focused on BV, *Chlamydia trachomatis* (*C. trachomatis*), and AV (Ceccarani et al., 2019). In addition, inflammation of the reproductive tract caused by bacterial flora disorders involving BV, AV, and *C. trachomatis* infection is closely related to adverse pregnancy outcomes and tumors (Sherrard et al., 2018). Other diseases, such as trichomoniasis (*Trichomonas vaginalis*), vulvovaginal candidiasis, and gonorrhea, have received less relevant research in this area and may become future research directions. Therefore, this section mainly discusses the interaction between the microorganisms, metabolites, and host immunity of three common RTIs: BV, *C. trachomatis* infection, and AV.

### BV

BV is the most common vaginal microbial disorder of women of childbearing age, and can lead to adverse obstetrics and gynecological outcomes such as infertility, miscarriage, premature rupture of membranes, and premature delivery (Workowski and Bolan, 2015; Baqui et al., 2019; Peebles et al., 2019). It also increases the risk of sexually transmitted infections (Shipitsyna et al., 2020). BV is characterized by an increase in the diversity of vaginal microbiota, a decrease in *Lactobacillus* spp. in the vagina, and an increase in BV-related anaerobic and microaerobes (Srinivasan et al., 2015). BV-related bacteria mainly include *Gardnerella*, *Atopobium*, *Mycoplasma*, *Megasphaera*, *Mobiluncus*, *Roseburia*, *Dialister.*, *Sneathia* and *Prevotella* spp. (McMillan et al., 2015; Ceccarani et al., 2019). However, flora analysis alone cannot distinguish between a normal vaginal environment and BV because *Atopobium* spp., *Prevotella* spp. and *Mycoplasma hominis* can also be detected in healthy people, therefore, the vaginal microenvironment needs to be analyzed in combination with metabolomics (Vitali et al., 2015).

BV is closely related to metabolites in the genital tract (Spiegel et al., 1980; Wolrath et al., 2002) (**Table 2**). The metabolites of amines, organic acids, short chain fatty acids (SCFAs), amino acids, nitrogenous bases and monosaccharides of BV patients are significantly different from those of healthy individuals (Vitali et al., 2015). Current studies suggest that metabolites better reflect the disease phenotype than microorganisms. Before disease symptoms appear, the appearance or disappearance of certain metabolites in the vagina has a positive or negative correlation with the metabolic function of certain microorganisms (Yeoman et al., 2013). Changes in maltose, kynurenine, nicotinate, malonate, acetate and nicotinamide adenine dinucleotide (NAD^+^) represent the occurrence of BV and can be used as metabolic biomarkers to distinguish BV from a healthy vagina (Vitali et al., 2015). When BV is cured, the metabolites associated with BV decrease significantly (Stanek et al., 1992; Srinivasan et al., 2015). In addition, genital tract metabolic analysis plays a prominent role in the diagnosis of BV (Watson and Reid, 2018). In 2015, McMillan et al. (2015) found that an increase in 2-hydroxyisovalerate and *γ*-hydroxybutyrate and a decrease in lactic acid and tyrosine in the vagina are the most sensitive and specific indicators for the diagnosis of BV. Therefore, not only the microbiota but also metabolites can be used as effective reference indicators for clinical diagnosis.

**Table 2:** Current existing articles analyzing the correlation between the genital tract flora and metabolites in women with vaginitis.

Year	Author	Population	Sample	CST		Metabolites
**BV**						
2019	(Ceccarani et al., 2019)	Healthy women (n = 21), women with BV (n = 20) and women with Chlamydia trachomatis infection (n = 20)	Vaginal swabs	CST-IV	1) Higher levels of organic acids (*i.e.*: formate, pyruvate, propionate, acetate, 2-hydroxyisovalerate), amines (*i.e.*: trimethylamine, putrescine), amino acids (*i.e.*: proline and alanine) and 5-aminopentanoate 2) Lower levels of lactate, 4-hydroxyphenylacetate, phenylalanine, pi-methylhistidine, glycine, isoleucine, leucine, tryptophan, aspartate, dimethylamine, and sarcosine	
2018	(Parolin et al., 2018)	Healthy women (n = 22), women with Chlamydia trachomatis infection (n = 20), and women with BV (n = 19)	Vaginal swabs	CST-IV	Higher levels of biogenic amines (methylamine, putrescine, trimethylamine, tyramine, desaminotyrosine), organic acids (succinate, malonate, 2-hydroxyisovalerate, and short-chain fatty acids) and alanine	
2015	(McMillan et al., 2015)	Pregnant women (n = 67) and non-pregnant women (n = 64)	Vaginal fluid	CST-IV	1) Organic acid: higher levels of 2-hydroxyisovalerate, γ -hydroxybutyrate, 2-hydroxyglutarate and 2-hydroxyisocaproate; lower levels of lactate 2) Amines: higher levels of tyramine, putrescine, and cadaverine 3) Amino acids: lower levels of tyrosine	
2015	(Srinivasan et al., 2015)	Women with BV (n = 40) and women with non-BV (n = 20)	Vaginal fluid	CST-IV	1) Amino acid: higher levels of cadaverine, pipecolate, tyramine, 4-hydroxyphenylacetate, 3- (4-hydroxyphenyl) propionate, tryptamine, citrulline and putrescine; lower concentrations of arginine, ornithine, spermine and dipeptides 2) Carbohydrates: higher levels of N-acetylneuraminate, galactose, threitol and succinate; lower levels of glucosamine, maltotriose, maltotetraose, maltopentaose, maltohexaose, lactate, fructose, and mannitol 3) NAD: lower levels of nicotinamide; higher levels of nicotinate 4) Lipids: higher levels of 12-hydroxyeicosatetraenoic acid, deoxycarnitine, 4-hydroxybutyrate and 13-hydroxyoctadecadienoic acid; lower levels of arachidonate, carnitine, ascorbic acid, acetylcarnitine, propionylcarnitine, butyrylcarnitine, glycerol and glycerol-3-phosphate	
2015	(Vitali et al., 2015)	BV-affected patients (n = 43) and healthy controls (n = 37)	Vaginal fluid	CST-IV	1) Amines: higher levels of tyramine, ethanolamine, trimethylamine, methylamine, cadaverine 2) Organic acids: higher levels of formate, malonate, succinate, pyruvate, acetate 3) Short-chain fatty acids: higher levels of propionate, butyrate, 2-hydroxyisovalerate 4) Amino acids: higher levels of proline; lower levels of tryptophan, phenylalanine, tyrosine, glutamate, isoleucine, leucine 5) Nitrogenous bases: higher levels of nicotinate, uracil; lower levels of NAD+, inosine 6) Sugars: higher levels of glucose; lower levels of maltose 7) Others: higher levels of urocanate, 2-aminoadipate, 3-methyl-2-oxovalerate; lower levels of kynurenine, sn-glycero-3-phosphocholine, sarcosine	
2013	(Yeoman et al., 2013)	Pre-menopausal women of reproductive age (n = 36)	Vaginal lavage fluid	CST-IV	1) Higher levels of putrescine, cadaverine, 2-methyl-2-hydroxybutanoic acid, hydroxylamine, glycolic acid, tetradecanoic acid, and butyrolactone 2) Lower levels of 2,3-hydroxypropyl-2-aminoethyl phosphate, cis-11-octadecanoic acid, and ribose-5-phosphate	
2012	(Gajer et al., 2012)	Reproductive age women (n = 32)	Vaginal swabs	CST-IV	1) Higher levels of succinate and acetate 2) Lower concentrations of lactate	
2002	(Wolrath et al., 2002)	Women of childbearing age with various lower genital tract disorders (n = 61)	Vaginal fluid	CST-IV	Higher levels of trimethylamine	
1980	(Spiegel et al., 1980)	Women with non-specific vaginitis (n = 53)	Vaginal fluid	CST-IV	1) Higher levels of succinate, acetate, butyrate, and propionate 2) Lower levels of lactate	
**Chlamydia trachomatis**						
2018	(Parolin et al., 2018)	Women with Chlamydia trachomatis infection (n = 20), healthy women (n = 22), and women with BV (n = 19)	Vaginal swabs	CST-III		Lower levels of tyramine, dimethylamine, cadaverine, succinate, valine, isoleucine, glycine, sarcosine, creatinine, 4-aminobutyrate
2019	(Ceccarani et al., 2019)	Healthy women (n = 21), women with BV (n = 20) and women with Chlamydia trachomatis infection (n = 20)	Vaginal swabs	CST-IV		Lower levels of lactate, certain amino acids and biogenic amines
**AV**						
2012	(Gajer et al., 2012)	Reproductive age women (n = 32)	Vaginal swabs	CST-IV		Higher levels of acetate and lactate

CST, Community state types; BV, bacterial vaginosis; AV, aerobic vaginitis.

The vaginal microbiota and metabolites of BV patients are also closely related to the clinical symptoms and signs of the host. The odor of vaginal secretions in patients with BV is related to the increase in tyramine, trimethylamine, cadaverine, and putrescine and the decrease in the aromatic substances 2 (5H)-furanone and 2-ethyl-4-methyl-1,3-dioxolane (Yeoman et al., 2013; Srinivasan et al., 2015; Vitali et al., 2015). Odor is also closely related to *Dialister* spp. (Yeoman et al., 2013; McMillan et al., 2015; Srinivasan et al., 2015). Thin and homogeneous secretions are positively related to cadaverine, and cadaverine is related to *Streptococcus* spp. and *Mycoplasma* spp. (Yeoman et al., 2013; Srinivasan et al., 2015). Clue cells are positively correlated with deoxycarnitine and pipecolate, while deoxycarnitine is positively correlated with BV-associated bacterium 1 (BVAB1), *Megasphaera* sp. type 2, and several *Prevotella* species (Srinivasan et al., 2015). Vaginal discharge is related to 2-methyl-2-hydroxybutanoic acid and *Mobiluncus* spp. (Yeoman et al., 2013). In addition, the metabolic pathways of amino acids, carbohydrates, NAD, and lipids in the vaginal flora of BV patients are active and are closely related to cellular life activities (Srinivasan et al., 2015; Ceccarani et al., 2019).Therefore, understanding the interaction between the flora and metabolites of BV patients provides a basis for understanding the molecular mechanisms of microbe-microbe and microbe-host interactions (Ilhan et al., 2019).

BV-related bacteria can activate the host’s genital tract immune response, but they do not cause obvious inflammatory symptoms such as redness, swelling, heat and pain (Smith and Ravel, 2017). The reason may be related to the influence of BV-related microorganisms and their metabolites on immunity. In 2019, Delgado-Diaz et al. (2019) found that the sustained action of organic acids, metabolites of the vaginal microbiota associated with BV, led to dysregulation of the immune response of cervical and vaginal epithelial cells *in vitro*. SCFAs can recruit and activate female reproductive tract innate immune cells, such as neutrophils and monocytes (Vitali et al., 2015). However, SCFAs can also inhibit the production of proinflammatory cytokines and affect the migration and phagocytic response of immune cells to regulate the immune response (Al-Mushrif et al., 2000). In addition, succinic acid produced by *Prevotella* spp. and *Mobiluncus* spp. in the genital tract can also inhibit leukocyte chemotaxis and regulate the immune response (Al-Mushrif et al., 2000; McMillan et al., 2015; Vitali et al., 2015). In 1985, Rotstein et al. (1985) demonstrated that succinic acid has the strongest chemotaxis inhibitory effect at pH 5.5 and at concentrations of 20–30 mM. Therefore, the current research has proven that the interaction between BV flora, metabolites and immunity is of great significance for understanding clinical symptoms and signs. However, more research on the interaction mechanism between immunity and metabolites in BV patients is needed to confirm the influence of metabolites on flora and immunity.

### Chlamydia trachomatis

In 2016, the World Health Organization announced the newest global *C. trachomatis* prevalence rate of 1.5–7% for women aged 15–49 years and an estimated 127 million new cases women worldwide that year (Organization, 2020). Most women infected with *C. trachomatis* are asymptomatic (Ceccarani et al., 2019). Approximately 10% of *C. trachomatis* infections will progress to PID without timely treatment, which will cause severe ectopic pregnancy, reproductive dysfunction and cancer (Workowski and Bolan, 2015; Idahl et al., 2020). Lactic acid is an important inhibitor of *C. trachomatis* infection (Gong et al., 2014). However, *L. iners* produces less lactic acid, so the microbiota dominated by *L. iners* increases the risk of *C. trachomatis* infection (Van Houdt et al., 2018). Similarly, BV also increases the risk of *C. trachomatis* infection due to a reduction in the lactate concentration (Shipitsyna et al., 2020). Therefore, *C. trachomatis* infection is greatly affected by lactic acid in the reproductive tract microenvironment.

The interactions among microorganisms, *C. trachomatis* and metabolites in the reproductive tract are closely related (**Table 2**). In 2016, Ceccarani et al. (2019) performed a combined metagenomic and metabolomics analysis on the vaginal secretions of non-pregnant Caucasians of childbearing age with risk factors for *C. trachomatis* infection. The study showed that *C. trachomatis* infection was dominated by *Lactobacillus* in most people, and *L. iners* was increased, and some patients had anaerobic bacteria as the dominant bacteria. Compared with healthy controls, women infected with *C. trachomatis* showed only slight changes in vaginal metabolites that were mainly manifested as a reduction in certain amino acids and biogenic amines (Ceccarani et al., 2019). In 2018, Parolin et al. (2018) studied the characteristics of vaginal microbes and metabolites in the case of *C. trachomatis* infection and found that vaginal valine, isoleucine, tyramine, cadaverine, and succinate in patients with *C. trachomatis* infection were significantly decreased compared with those in healthy controls, indicating that *C. trachomatis* may use nitrogen as the first nutrient source or that *C. trachomatis* may affect the nitrogen metabolism of infected host cells. There is a correlation between the vaginal microbiome, metabolites, and genital symptoms of *C. trachomatis* infection (Parolin et al., 2018). More than half of *C. trachomatis*-infected patients are completely asymptomatic, while symptomatic patients mainly manifest with abnormal vaginal discharge, dyspareunia, dysuria, and abnormal bleeding. The concentration of 4-aminobutyrate is significantly different between asymptomatic and symptomatic women with *C. trachomatis* infection. However, all asymptomatic women have *L. crispatus* as the dominant bacteria in the vagina, and only half of symptomatic women have *L. crispatus* as the dominant bacteria (Parolin et al., 2018). Therefore, *C. trachomatis* infection is related to the genital tract flora, metabolites and clinical symptoms. However, the effect of 4-aminobutyrate on host immunity against *C. trachomatis* infection needs further study.

There are complicated interactions between *C. trachomatis*, microorganisms, genital tract metabolites and immunity (Ziklo et al., 2016b). Epithelial cells and immune cells infected by *C. trachomatis* can secrete several proinflammatory cytokines and chemokines to eliminate pathogen infection (Rasmussen et al., 1997; Johnson, 2004; Brunham and Rey-Ladino, 2005). Interferon (IFN)-γ is an important factor that inhibits the reproduction of *C. trachomatis* (Shemer and Sarov, 1985). The ability to synthesize tryptophan in the IFNγ-rich infection microenvironment is an important virulence factor of the genital *C. trachomatis* serovars (Aiyar et al., 2014). IFN-γ mediates the activation of host indoleamine 2,3-dioxgenase (IDO), leading to the consumption of tryptophan necessary for the growth of *C. trachomatis* and inhibiting the growth of *C. trachomatis* (Beatty et al., 1994; Aiyar et al., 2014; Olive and Sassetti, 2016; Molenaar et al., 2018). The tryptophan needed for the growth of *C. trachomatis* is reduced, and *C. trachomatis* forms a static state (Byrne et al., 1989). It is known that BV infection increases the risk of *C. trachomatis* infection. BV-related bacteria, such as partial *Prevotella species*, can produce indole (Romanik et al., 2007; Sasaki-Imamura et al., 2011), which is increased in the vaginal discharge of BV patients (Lewis et al., 2014). *C. trachomatis* in the genital tract can use indole produced by microorganisms as a substrate to activate alternative tryptophan synthesis pathways- the *trpA*, *trpB* and *trpR* genes, synthesize tryptophan, and make *C. trachomatis* evade the clearance of IFN-γ in the genital tract (Fehlner-Gardiner et al., 2002; Wood et al., 2003; Ziklo et al., 2016b). However, in patients who are coinfection with BV and *C. trachomatis*, the inhibitory response to IFN-γ is not exactly the same, which may be related to the level of indole in the vaginal microenvironment (Lewis et al., 2014). In addition, the low oxygen environment formed under BV may result in insufficient energy supply for the IFN-*γ* signaling pathway, which further reduces its function (Roth et al., 2010). IFN-*γ* induces the production of nitric oxide and further inhibits the growth of *C. trachomatis* (Agrawal et al., 2011). The most recent *in vitro* experiments have confirmed that *C. trachomatis* induces the expression of ornithine decarboxylase (ODC), deprives the inducible nitric oxide synthase (iNOS) substrate arginine, and actively promotes polyamine synthesis while downregulating iNOS expression and inhibiting the activity of iNOS to reduce nitric oxide production in the host and further escape the host’s innate immunity (Abu-Lubad et al., 2014; Olive and Sassetti, 2016). Studies have shown that the amino acids and sugars in the environment are critical to the ability of *C. trachomatis* to infect (Harper et al., 2000). However, the detailed metabolic and immune interaction mechanisms still need further study. After *C. trachomatis* escapes the host’s immunity, the host’s immune surveillance is reduced, and the environment is conducive to the growth of *C. trachomatis*, which is reactivated (Belland et al., 2003). After *C. trachomatis* reinfection or chronic infection, T helper (Th)1-, Th2- and Th17-type cells are triggered to mediate tissue destruction, fibrosis, and scarring, further leading to the progression of PID and its sequelae (Ziklo et al., 2016a; Molenaar et al., 2018).

### AV

The incidence of AV in women of childbearing age is approximately 10% (Donders et al., 2017). Significantly different from BV patients and those with a normal flora, AV patients have increased aerobic bacteria or enterococci, such as *Escherichia coli*, *Streptococcus agalactiae*, *Staphylococcus aureus*, *Staphylococcus epidermidis*, *Streptococcus anginosus*, and *Enterococcus faecalis*, in the vagina (Donders et al., 2017; Tao et al., 2019; Wang et al., 2020). Studies have also shown that BV-related bacteria often appear in the vaginal flora of AV patients, which may be related to the symbiotic relationship between BV- and AV-related bacteria in the state of flora disorder (Wang et al., 2020). Different from the clinical symptoms and signs of BV, AV mainly manifests as foul and yellow purulent discharge, but similar to BV, it easily causes adverse obstetrics and gynecology complications that may be related to bacterial ascending infection (Donders et al., 2017).

It is known that AV-related bacteria and their metabolites are involved in the host’s inflammatory state and immune response, but their correlation with host disease phenotypes and diagnostic applications have not yet been studied. Previous studies have found that when *Streptococcus* sp. increase in the vaginal secretions of non-pregnant women, acetate also increases (Gajer et al., 2012) (**Table 2**). Acetate is a SCFAs that directly activates the host’s immune-inflammatory pathway and promotes the expansion of T-regulatory (Treg) cells (Olive and Sassetti, 2016; Postler and Ghosh, 2017). However, the detailed mechanism by which acetic acid is produced by *Streptococcus* sp. and genital tract immunity still needs to be studied.

AV patients mainly present with a local immune imbalance in the reproductive tract caused by pathogens (Benner et al., 2018). In 2020, Budilovskaya et al. (2020) found that the expression of IL1β, IL-6, IL-8, IL10, tumor necrosis factor-α (TNFα) and CD68 messenger ribose nucleic acids (mRNAs) in AV patients was significantly increased, and this change was related to itching or burning as well as increases in leukocytes and parabasal epithelial cells under the microscope (Smith and Ravel, 2017). Purulent vaginal discharge and vaginal redness may be related to the toxic effect of the virulence gene *sag* of *Streptococcus anginosus* on epithelial cells, leading to epithelial cell lysis (Tao et al., 2019).

The molecular mechanism of inflammatory genital tract symptoms in AV patients may be related to the interaction of metabolism and immunity. Nitric oxide plays an important role in host resistance to pathogens. Nitric oxide is synthesized by iNOS in inflammatory cells (Richardson et al., 2006; Jones et al., 2010). After the cells secrete nitric oxide, they can kill pathogens directly beside the inflammatory cells. However, *Staphylococcus aureus* can evade the host nitric oxide response by changing metabolism (Olive and Sassetti, 2016). *Staphylococcus aureus* induces the expression of flavohaemoglobin (Hmp) through the SrrAB system, quickly and enzymatically hydrolyzes nitric oxide, and resists the host’s inhibitory effect on pathogens (Richardson et al., 2006); at the same time, *Staphylococcus aureus* upregulates L-lactate dehydrogenase 1 (Ldh1), enabling it to survive lactic acid fermentation under aerobic and anaerobic conditions (Richardson et al., 2008). The virulence of *Staphylococcus aureus* requires hexose produced by glycolysis, and an increase in the glucose concentration will enhance the resistance of pathogens to nitric oxide and subsequently the host immune response (Vitko et al., 2015; Olive and Sassetti, 2016). Polyamines are toxic to *Staphylococcus aureus*. *Staphylococcus aureus* strains with arginine catabolic mobile element (ACME) encode the acetyltransferase SpeG, which makes the strains resistant to polyamines and facilitates colonization in host cells (Diep et al., 2008; Olive and Sassetti, 2016). Pathogens evade the killing effect of the host’s immune system, facilitating the colonization of pathogens in the host’s reproductive tract. The colonization of toxic shock syndrome toxin-1 (TSST-1) *Staphylooccus aureus strains* will increase the production of proinflammatory cytokines and chemokines in human vaginal epithelial cells, further destroying the mucosal barrier and increasing the penetrating effect of TSST-1, leading to severe symptoms and signs of vulvovaginitis (Pereira et al., 2013). This also explains why vaginal inflammation in AV is more serious than that in BV. However, previous studies have mainly focused on of BV-related bacteria, and there are few studies on AV-related bacteria, metabolites, and immunity. Future research may reveal the significance if it is used as a future research direction.

## Normal Pregnancy

Unlike non-pregnant women, healthy pregnant women are affected by estrogen-progesterone, and the vaginal microflora tends to be stable from the first trimester to the third trimester, that is, the low richness and low diversity dominated by *Lactobacillus* spp. inhibits the growth of CST IV pathogenic bacteria such as *Gardnerella vaginalis, Atopobium vaginae, Sneathia amnii*, *Prevotella Bivia*, and *Prevotella cluster 2* (MacIntyre et al., 2015; Brown et al., 2018; Serrano et al., 2019). The vaginal flora during pregnancy is less transformed, mostly between *Lactobacillus* species (Romero et al., 2014; Serrano et al., 2019). Pregnant women with CST I as the dominant bacteria have the most stable vaginal flora throughout pregnancy, followed by those with CST V, CST II, and CST IV (MacIntyre et al., 2015). The vaginal microbiota during the third trimester is similar to that of non-pregnant women. One week after delivery, estrogen decreases, and glycogen-supported *Lactobacillus* spp. also decreases sharply. The stability and compliance of the vaginal flora decreased significantly, and the diversity increases, especially that of CST IV, which leads to disorders of the postpartum vaginal flora and even postpartum endometritis and puerperal morbidity (DiGiulio et al., 2015; MacIntyre et al., 2015). There are also microflora in the placenta and amniotic fluid of women who experience normal-term delivery (Collado et al., 2016; Nuriel-Ohayon et al., 2016). Some studies suggest that placental bacteria may be derived from oral flora, mainly non-pathogenic symbiotic flora such as *Firmicutes*, *Tenericutes*, *Proteobacteria*, *Bacteroidetes*, and *Fusobacteria* phyla (Aagaard et al., 2014). The origin of the placental microbiota is also related to the migration of the intestinal flora to the fetus-placenta interface, which promotes colonization of the fetus after birth (Collado et al., 2016). However, some studies indicate that there are no microorganisms in the normal placenta and amniotic fluid, which may be caused by the contamination of laboratory reagents or equipment or by different methods used to obtaining specimens (Leiby et al., 2018; Lim et al., 2018; De Goffau et al., 2019).

Analysis of the metabolic characteristics of the vaginal microbiota revealed that the microbial metabolic activity in the first trimester is the highest to adapt to changes in pregnancy (Serrano et al., 2019) (**Table 3**). As pregnancy progresses, the vaginal microbiota tends to become stable, and its metabolic capacity tends to be simplified, mainly reflected in the low activity of carbohydrate metabolism, cell wall/membrane biochemical pathways, protein synthesis pathways, and nucleic acid metabolism pathways (Serrano et al., 2019). Another study also found that the carbohydrate metabolism and lipid metabolism of cervicovaginal secretions in full-term women were downregulated during the second and third trimesters (Ghartey et al., 2015). Carbohydrate metabolism was significantly downregulated, especially in the third trimester of pregnancy, and was related to the large amount of glycogen deposition and metabolization into lactic acid in a highly estrogen state (Ghartey et al., 2015). This change is conducive to the colonization of lactobacilli in the host reproductive tract and maintains the necessary acidic pH in the “healthy” reproductive tract. It also helps maintain the integrity of the cervix and is related to a reduction in adverse pregnancy outcomes. In women who give birth at term, the lipid metabolism of cervicovaginal secretions is significantly reduced in the third trimester, which may be related to the acidic environment inhibiting the growth of pathogenic bacteria, and the antimicrobial component of cervicovaginal secretions, methyl-4-hydroxybenzoate, increases by approximately 8.8 times from the second to the third trimester, helping maintain a stable vaginal microenvironment (Ghartey et al., 2015). The metabolic pathways of amniotic fluid and placental flora are mainly involved in membrane transport, carbohydrate metabolism, amino acid metabolism and energy metabolism, which are closely related to the life activities of the fetal placenta (Aagaard et al., 2014; Collado et al., 2016). Therefore, the metabolic function of the genital tract flora during pregnancy is of great significance for maintaining pregnancy stability.

**Table 3:** Current existing articles analyzing the correlation between the genital tract flora and metabolites in pregnant women.

Year	Author	Population	Sample	CST/microorganisms	Metabolites
**Normal pregnant women**					
2016	(Prince et al., 2016)	Women who delivered at term (n = 27), women who delivered preterm (n = 44)	Placental membranes swabs	*Bradyrhizobium* spp., *streptococcus thermophilus*	Term cohorts: lower levels of the amino sugar and nucleotide sugar metabolism, butanoate metabolism, riboflavin metabolism, and amino-benzoate degradation
2015	(McMillan et al., 2015)	Pregnant women (n = 67) and non-pregnant women (n = 64)	Vaginal fluid	CST-I	Similar with non-pregnant women : Higher levels of succinate
				CST-IV	Similar with non-pregnant women : 1) Organic acid: higher levels of 2-hydroxyisovalerate, *γ*-hydroxybutyrate, 2-hydroxyglutarate and 2-hydroxyisocaproate; lower levels of lactate 2) Higher levels of amines: tyramine, putrescine, and cadaverine 3) Lower levels of amine precursors: tyrosine, lysine, ornithine
**Preterm birth**					
2016	(Prince et al., 2016)	Women who delivered at term (n = 27), women with spontaneous preterm birth (n = 44)	Placental membranes swabs	*Lactobacillus crispatus*, *Acinetobacter johnsonii*	Preterm cohorts: higher levels of pentose phosphate pathway, glycerophopholipid metabolism, and biosynthesis of the siderophore group non-ribosomal peptides
2014	(Aagaard et al., 2014)	Women who delivered with preterm birth (n = 16), women with remote antenatal infection (n = 16), and controls (n = 16)	Placenta tissue	*Burkholderia spp.*	1)Higher levels of methane metabolism, isoquinoline alkaloid biosynthesis, and glycine/serine/threonine metabolism 2) Lower levels of biotin metabolism and glycosylphosphatidylinositol anchor pathways

CST, Community state types.

In normal pregnancy, the mother has increased immune tolerance to fetal-expressed paternal antigens through “extended-self” antigens to maintain the growth of the fetus in the body (Bromfield et al., 2017; Deshmukh and Way, 2019). Maternal forkhead box P3 (FOXP3) Treg cells expand locally at the maternal-fetal interface and expand systemically during pregnancy to maintain allogeneic fetal tolerance (Agostinis et al., 2019; Deshmukh and Way, 2019; Ghaemi et al., 2019). Metabolites are closely related to host immunity during pregnancy. In humans, the metabolism of L-arginine is related to the temporary suppression of the maternal immune response during pregnancy (Kropf et al., 2007). The activity of arginase expressed in the full-term placenta of pregnant women increases significantly, and the high enzyme activity leads to a decrease in its substrate L-arginine, which in turn induces the downregulation of T-cell receptor (TCR) associated *ζ*-chain (CD3*ζ*) and the hyporesponsiveness of functional T cells (Ismail, 2018). IDO also uses a similar approach to silence T cells to induce and maintain immune tolerance (Kropf et al., 2007). The normal reproductive tract flora plays an important role in the establishment and consolidation of mother-placental-fetal immunity to resist the interference of external pathogenic bacteria (Mei et al., 2019). Studies have demonstrated a correlation between *Bacteroides* species and TCR*γδ*+ T cells (participating in mucosal immunity) (Ghaemi et al., 2019). The microbiota can induce the accumulation of Treg cells, which are essential for maintaining immune tolerance, timely endometrial receptivity, and correct placental implantation (Benner et al., 2018). However, dysbiosis in the reproductive genital tract can lead to immune disorders and participate in the occurrence of adverse pregnancy outcomes (Smith and Ravel, 2017). Therefore, a comprehensive interpretation of the reproductive tract microbes, metabolism, and immunity during normal pregnancy provides a reference for discovering the causes and mechanisms of adverse pregnancy outcomes (Wang et al., 2016).

## Pregnancy-Related Adverse Outcomes

### Spontaneous Abortion and Infertility

Statistics from the Centers for Disease Control in the United States showed that among married women aged 15–44 years, 6% had infertility and 12% had impaired fecundity, and the incidence increased yearly (Prevention, 2019). RTI is a risk factor leading to reproductive dysfunction (such as infertility, miscarriage, and repeated fertility failures), which in turn leads to a clinical pregnancy rate of only 29.7–43.3% with embryo transfer technology (Baker et al., 2010; Franasiak et al., 2016; Koedooder et al., 2019). For example, the prevalence of BV in infertile women is 19–28%, while the clinical pregnancy success rate is only 8% (Haahr et al., 2016; Bracewell-Milnes et al., 2018). This observation may be related to abnormal vaginal microbiota and pelvic pathogens (such as *C. trachomatis*) ascending to the upper genital tract through the cervix, leading to PID and reduced fertility (Witkin et al., 1995; Franasiak et al., 2016; Haahr et al., 2016).

The normal reproductive tract flora provides a favorable environment for embryo implantation, which can increase the success rate and live birth rate of *in vitro* fertilization-embryo transfer (IVFET) (Hyman et al., 2012; Sirota et al., 2014; Koedooder et al., 2019). The genital tract microbiota in females with fertility disorders is mainly manifested as a decrease in *Lactobacillus* spp., an increase in non-*Lactobacillus* spp., a high concentration of *Candida* spp., and an increase in the prevalence of asymptomatic BV (Franasiak et al., 2016; Babu et al., 2017; Campisciano et al., 2017; Wee et al., 2018; Koedooder et al., 2019). The live birth rate will be reduced if harmful bacteria, such as *Gardnerella vaginalis*, *Atopobium vaginae*, *Acidovorax* spp., *Enterococcus* spp., and *Streptococcus* spp., are found in the lower reproductive tract (Hyman et al., 2012; Haahr et al., 2016; Wee et al., 2018; Koedooder et al., 2019). Studies have shown that before spontaneous abortion, endometrial aspiration fluid has higher bacterial diversity and lower *Lactobacillus* abundance than before a healthy pregnancy (Moreno et al., 2020). The presence of CST IV microbiota in the endometrium is related to a significant reduction in the incidence of implantation, pregnancy, and continuous pregnancy (Moreno et al., 2016). In 2016, Verstraelen et al. (2016) performed endometrial biopsy on 19 women with fertility disorders (infertility, repeated implantation failures, and repeated miscarriages) and found that 90% of women with fertility disorders mainly had *Bacteroides* phylum as the dominant bacteria in their endometrium. In addition, when *Gardnerella* and *Streptococcus* genra are detected in the endometrium, they have a particularly adverse effect on reproductive outcomes (Moreno et al., 2016). The endometrium microbiota may be carried by sperm and affect the microbial composition of the female reproductive tract (Koedooder et al., 2019). For example, when the detection rate of *Mycoplasma hominis, Neisseria genus, Klebsiella genus* and *Pseudomonas genus* in semen increases, it is not only related to a low sperm concentration, abnormal sperm morphology, high semen viscosity, and oligospermia but also indirectly leads to a decline in female fertility (Ahmadi et al., 2017; Monteiro et al., 2018). Microorganisms also colonize in the follicular fluid, and the low success rate of embryo transfer is related to the colonization of *Propionibacterium* spp. and *Streptococcus* spp. in the follicular fluid (Pelzer et al., 2013). Therefore, the normal genital tract flora is of great significance to the maintenance of fertility.

Endometrial receptivity and follicle quality in people with reproductive disorders are closely related to metabolites in the reproductive tract. Lipid homeostasis is essential for maintaining health (Braga et al., 2019; Hernandez-Vargas et al., 2020). In 2019, Braga et al. (2019) analyzed the lipid metabolism of the endometrial secretions taken from patients with IVFET cycles before transplantation and found that phosphoethanolamine, phosphatidic acid, diacylglycerol, triacylglycerol, glycosyl diacylglycerol, phosphatidylcholine, neutral sphingolipid, and lysophosphatidylglycerol are possible biomarkers of endometrial receptivity and are associated with implantation failure (Altmäe et al., 2014). Follicular fluid is the microenvironment for the growth of oocytes, and the metabolism of follicular fluid indirectly affects the growth and development of oocytes (Bracewell-Milnes et al., 2017). In 2019, Song et al. (2019) conducted a targeted metabolomics analysis of the follicular fluid of patients with recurrent spontaneous abortion after IVFET treatment and found that eight metabolites, namely, dehydroepiandrosterone, lysophosphatidylcholine (lysoPC) (16:0), lysoPC(18:2), lysoPC(18:1), lysoPC(18:0), lysoPC(20:5), lysoPC(20:4), and lysoPC(20:3) were upregulated in the recurrent abortion group, and 10 metabolites, namely, phenylalanine, linoleate, oleic acid, docosahexaenoic acid, lithocholic acid, 25-hydroxyvitamin D3, hydroxycholesterol, 13-hydroxy-alpha-tocopherol, leucine, and tryptophan were downregulated. The above indicators can also predict the success rate of transplantation. Therefore, it is very meaningful to analyze metabolites in people with reproductive disorders. However the metabonomic analysis of cervicovaginal secretions in women with fertility disorders still needs more research to fully prove the role of metabolism in fertility disorders and the interaction between immunity and the flora.

Fertility dysfunction may be related to the destruction of immune tolerance caused by a decline in the number and function of Treg cells (Deshmukh and Way, 2019).Through endometrial biopsies of women with infertility in the mid-secretory phase of the menstrual cycle, it was found that the expression of Foxp3 mRNA was reduced, suggesting that the differentiation of uterine T cells into a Treg cell phenotype is impaired, which may lead to reduced endometrial receptivity (Jasper et al., 2006). Moreover, immunoglobulin-like transcript 4+ (ILT4+) DCs may be involved in the process of recurrent miscarriage and recurrent implantation failure induced by Foxp3+ Treg cells (Liu et al., 2018). A reduction in maternal Treg cell inhibitory ability caused by microbial infection can also cause placental inflammation, leading to the release and activation of fetal-specific maternal CD8+ T cells, which infiltrate the decidua and lead to abortion (Deshmukh and Way, 2019). The microbiota is important for basic CCL2 (monocyte chemotactic protein-1, MCP-1) secretion to control the homeostasis of plasmacytoid DCs, macrophage recruitment and polarization, and local T cell balance (Sierra-Filardi et al., 2014; Swiecki et al., 2017). DCs are a key regulator of immune tolerance during pregnancy. Patients with elevated dehydroepiandrosterone and dehydroepiandrosterone sulfate (DHEAS) in the follicular fluid have DC damage, which can cause infertility or spontaneous abortion by causing the abnormal immunity of oocytes or embryos (Song et al., 2019). When combined with a bacterial flora disorder, it may aggravate the dysfunction of DCs, and reproductive dysfunction is likely. It is known that plasma tryptophan metabolism is closely related to abortion (Fei et al., 2016). While BV-related bacteria are involved in the metabolism of tryptophan and a variety of amino acids (Srinivasan et al., 2015), it is necessary to further study whether genital tract bacteria and their metabolites cause an imbalance of immune tolerance, affect plasma metabolite levels and participate in the occurrence of reproductive dysfunction. Future research should focus on the local immune effects of genital tract flora and metabolites on people with reproductive dysfunction as the main research direction to explore the impact of the three interactions on reproductive disorders.

### Preterm Birth

Every year, 15 million babies are born premature worldwide, accounting for approximately 11% of the live birth population (Blencowe et al., 2012). Preterm birth caused by ascending genitourinary tract infection accounts for 40–50% of all preterm births (Goldenberg et al., 2008). CST-IV vaginal microflora is closely related to premature delivery (DiGiulio et al., 2015; Dunlop et al., 2015; Workowski and Bolan, 2015; Brown et al., 2018; Han et al., 2019). Additionally, studies have confirmed that pregnant women with BV have an increased risk of premature birth (Callahan et al., 2017; Anahtar et al., 2018; Chu et al., 2018; Fettweis et al., 2019; Serrano et al., 2019). Preterm birth is the second leading cause of neonatal death (Liu et al., 2016). Premature babies are prone to diabetes, chronic inflammation and cardiovascular disease in the long term (Snyers et al., 2020). Therefore, the prevention of premature birth is the top priority of medical work.

The Human Microbiome Project Multi-Omic Microbiome Study showed that *L. crispatus* decreased and that BVAB1, *Sneathia amnii*, TM7-H1 (BVAB-TM7), and partial *Prevotella* species increased in the first and second trimesters of women who deliver prematurely, thus, these factors can be used as markers for predicting preterm birth (Fettweis et al., 2019). Studies have also shown that the colonization of vaginal *Streptococcus agalactiae* and *Klebsiella pneumonia* in the second trimester is significantly associated with late miscarriage and very premature delivery (before 28 weeks) (Son et al., 2018; Koedooder et al., 2019). Changes in the cervicovaginal flora of women who deliver prematurely greatly alter the metabolome and are involved in premature cervical remodeling (**Table 3**). Ghartey et al. (2015; 2017) found that women with symptoms of preterm birth and eventually spontaneous preterm birth (sPTB) have significant changes in cervicovaginal metabolites. Lipid metabolism and carbohydrate metabolism in the cervicovaginal secretions of women who deliver prematurely are significantly upregulated, and peptide levels are significantly reduced (Ghartey et al., 2015; Ghartey et al., 2017). Upregulation of lipid and carbohydrate pathways is associated with positive energy utilization and may be related to early cervical remodeling and sPTB microbiota utilization (Ghartey et al., 2015; Ghartey et al., 2017). A decrease in dipeptides may reflect the decreased level of proteolysis in women who deliver prematurely and changes in the activities of proteases and are associated with asymptomatic sPTB (Ghartey et al., 2015). In addition, the level of N-acetylneuraminate in the cervix of women who deliver prematurely increased significantly (by 4.9 times), which may be related to the increased affinity of cells for infection and participate in host immunity (Ghartey et al., 2015).

Embryo development and growth depend to a large extent on placental function, and the placental microbiome may affect fetal and pregnancy outcomes. Chorioamnionitis and intrauterine infection are closely related to premature delivery (Chu et al., 2018). However, the specific source of infection may be the ascending infection of BV bacteria (Fettweis et al., 2019), the ascending carrying of sperm (Svenstrup et al., 2003), the colonization of endometrial bacteria (Cowling et al., 1992; Chu et al., 2018), the retrograde infection of salpingitis, and the blood-borne infection of oral bacteria (Chu et al., 2018). Studies have shown that the bacteria in the uterus of women who deliver prematurely are mainly derived from vaginal bacteria, such as *Burkholderia* taxa, which is significantly enriched in the placenta (Goldenberg et al., 2000; Aagaard et al., 2014). The metabolic enrichment of the lipopolysaccharide biosynthetic pathway of the microbiota in the placenta may be related to the expansion and reproduction of the microbiota (Aagaard et al., 2014). The premature birth rate of women with bacteria detected in amniotic fluid is higher, and the metabolomics of the amniotic fluid of women who deliver prematurely are significantly altered, contributing to the initiation of preterm birth (Menon et al., 2014; Collado et al., 2016). Studies have shown that there are flora on the fetal membranes and that the composition of the fetal membranes is closely related to the degree of inflammation of chorioamnionitis (Prince et al., 2016). Analysis of the metabolic function of the fetal membrane microbiome revealed that a reduction in the pentose phosphate pathway and glycerophospholipid metabolism is related to chorioamnionitis, and a reduction in glycerophosopholipid metabolism will lead to an increase in the production of arachidonic acid, which is related to inflammation and prostanoid synthesis and is involved in premature birth (Prince et al., 2016).

Inflammation and antimicrobial peptide reactions involved in certain vaginal microorganisms play a role in destroying and invading cervical mucus plugs or amniotic membranes and ultimately trigger proinflammatory reactions, leading to premature delivery (Goldenberg et al., 2000; Yarbrough et al., 2015; Smith and Ravel, 2017; Strauss et al., 2018). It is known that *Gardnerella vaginalis* ascends to infect the amniotic membrane and irritate the cervix, leading to premature delivery. *Gardnerella vaginalis* may activate the NACHT, LRR and PYD domains-containing protein 3 (NLRP3) inflammasomes through monocyte NLRs; then, NLRP3 binds to and cleaves caspase-1, induces IL-1β, IL-18, and TNF-α secretion, and ultimately leads to premature delivery (Vick et al., 2014). Metabolites are involved in the occurrence and development of preterm labor. When *L. iners* and BV-related microorganisms are increased in the vagina, the ratio of D-type/L-type lactic acid decreases, and matrix metalloproteinase (MMP-8) increases (Witkin et al., 2013; MacIntyre et al., 2015). This process eventually leads to premature cervix maturation and ascending infection of the amniotic membrane and thus, premature delivery (Yoon et al., 2001). Four proinflammatory cytokines, eotaxin, IL-1β, IL-6 and MIP-1β, are increased significantly in the vagina of women who deliver prematurely (Fettweis et al., 2019). BVAB1, *Sneathia amnii*, TM7-H1, *Prevotella timonensis*, and *Prevotella buccalis* are closely related to the levels of cytokines in vaginal secretions (Fettweis et al., 2019). Both BVAB1 and TM7-H1 can produce pyruvate, acetate, L-lactate and propionate. These SCFAs reduce antimicrobial activity and promote the production of host proinflammatory cytokines and are also involved in the occurrence of preterm labor (Fettweis et al., 2019). Therefore, there is a close correlation between the microbiota, metabolites and host immune status in reproductive tracts of women who deliver prematurely, and future researchers need to study the relevant pathogenesis. However, the relationship of microbial metabolites in the reproductive tract, preterm delivery immunity and the onset of preterm delivery still needs further exploration.

## Gynecological Oncology

Dysbiosis is related to tumor carcinogenicity (Ilhan et al., 2019; Scott et al., 2019). An imbalance in specific microorganisms can lead to host epithelial barrier dysfunction, genome integration, genotoxicity, inflammatory activation, immune abnormalities and metabolic abnormalities, creating a microenvironment that allows tumor growth and further leading to the occurrence, development and/or transfer of gynecological malignancies (Scott et al., 2019; Laniewski et al., 2020). Among them, inflammation is the central feature of carcinogenesis and the main carcinogenic mechanism related to cancer (Scott et al., 2019). Microbial virulence factors can induce chronic inflammation in host tissues, stimulate cell proliferation, cause cell proliferation disorders, and combine with the failure of cell apoptosis to ultimately lead to a malignant phenotype (Scott et al., 2019; Laniewski et al., 2020). Metabolic changes in cancer are the core of tumorigenesis and phenotypic changes (Buckendahl et al., 2011; Zhang et al., 2012; Turkoglu et al., 2016; Hopkins and Meier, 2017; Icard et al., 2018). Human microorganisms are also involved in the formation of carcinogenic metabolites and even exert genotoxicity to cause host deoxyribonucleic acid (DNA) damage and participate in tumor carcinogenicity (Kassie et al., 2001; Scott et al., 2019). Flora disorders can also destroy the host-based anticancer immune monitoring to promote tumor development and progression (Di Pietro et al., 2018; Klein et al., 2019; Scott et al., 2019; Laniewski et al., 2020). Therefore, the balance of the microenvironment of the reproductive tract has a positive effect on maintaining the stability of the microbiota and antitumor effects.

### Cervical Intraepithelial Lesions and Cervical Cancer

Cervical cancer (CC) is the most common human papillomavirus (HPV)-related malignant tumor and the fourth most common malignant tumor in women worldwide. In 2018, there were an estimated 570,000 new cases and 311,000 deaths from this disease (Bray et al., 2018). Approximately 85-90% of high-risk HPV infections can be cleared spontaneously, and only 10-15% that persist lead to cervical intraepithelial neoplasia (CIN) and invasive cervical cancer (ICC). HPV-16 and HPV-18 are the main pathogens of CC (Chase et al., 2015). The surface of the cervical mucosa is susceptible to environmental influences. When dysbiosis occurs, the local cervicovaginal microenvironment may promote the progression of malignant tumors together with HPV (Ma et al., 2014; Laniewski et al., 2018; Chorna et al., 2020).

A decrease in *Lactobacillus* spp. and an increase in vaginal pH are closely related to HPV infection, cervical lesions and CC (Laniewski et al., 2018; Ilhan et al., 2019; Laniewski et al., 2020). Cervical squamous intraepithelial lesions or CC also increase the diversity of the vaginal flora, limited not only to BV-related microorganisms but also to non-BV bacteria, such as *Streptococcus agalactiae*, *Clostridium* spp., *Pseudomonadales* order, and *Staphylococcus* spp. (Klein et al., 2019; Laniewski et al., 2020). An increase in the CIN stage is also related to an increase in the diversity of the vaginal microbiota, suggesting that microorganisms play a role in the regulation of persistent viral infection and disease progression (Van Ostade et al., 2018). Compared with patients with low-grade squamous intraepithelial lesions (LSILs), *Sneathia sanguinegens*, *Anaerococcus tetradius* and *Peptostreptococcus anaerobius* in patients with high-grade squamous intraepithelial lesions (HSILs) are more enriched in the vagina, and *Mycoplasmatales* order, *Pseudomonadales* order, and *Staphylococcus* spp. are more enriched in the cervix (Mitra et al., 2015; Klein et al., 2019; Laniewski et al., 2020). Another study found that *Sneathia* spp. and *Fusobacterium* spp. exist only in women with cervical lesions or cancer but not in women without lesions (Audirac-Chalifour et al., 2016). Therefore, the presence of *Sneathia* in the vaginal microbiome may be a characteristic microorganism of cervical lesions and CC (Laniewski et al., 2018; Laniewski et al., 2020). Analysis of the metabolic pathways of the bacterial flora in patients with CC showed that the peptidoglycan biosynthesis (ko00550) pathway is significantly enriched (Kwon et al., 2019). Research has also found that the bacterial cell wall peptidoglycan is not only essential for maintaining the overall antiosmotic pressure of the bacteria to ensure cell survival but also participates in the occurrence of inflammation, affecting the function of host neutrophils and the innate immune response. Therefore, cervical microbes may promote the development of CC and precancerous lesions by acting as a modulators of host inflammatory pathways.

The vaginal metabolism characteristics of HPV-infected and uninfected patients are different, and the vaginal CST status drives the metabolic characteristics of HPV-infected patients (Borgogna et al., 2020) (**Table 4**). In vaginal CST III, HPV-infected women have higher levels of biogenic amines than HPV uninfected women (Borgogna et al., 2020). In CST IV, HPV-infected women have lower concentrations of glutathione (GSH), oxidized glutathione (GSSG), glycogen, and phospholipid-related metabolites than uninfected women. There are also differences in the metabolic characteristics of HPV infection, cervical lesions, and CC. Several researchers conducted a study on the metabolome of cervicovaginal secretions in HPV-mediated cervical tumors and found that compared with HPV-negative group, HPV-positive group, and cervical lesion group, the number and diversity of cervical vaginal metabolites in CC patients were increased (Ilhan et al., 2019). Compared with the HPV-negative group, the HPV-positive, LSIL and HSIL groups had fewer amino acids, and their metabolites in cervical and vaginal secretions and the subpathways and depletion levels under the amino acid superpathway were different (Ilhan et al., 2019; Laniewski et al., 2020). In addition, in the vulvovaginal secretions of patients with CC, volatile organic compounds, such as alkanes, and methylated alkanes are different from those of healthy women, which may be related to the oxidation of cell membrane lipids and proteins during the carcinogenic process and the production of volatile organic compounds (Rodriguez-Esquivel et al., 2018; Ilhan et al., 2019). These metabolites can be used as potential biomarkers for CC. In addition, the unique metabolic characteristics in the cervicovaginal microenvironment can assist in the diagnosis and differentiation of health, HPV infection (Borgogna et al., 2020), HSILs, LSILs, and CC (Ilhan et al., 2019). For example, long chain fatty acids, ketone bodies, steroids, ceramides, and plasmalogens can distinguish individuals with ICC from those with HPV (−).

**Table 4:** Current existing articles analyzing the correlation between the genital tract flora and metabolites in women with cervical intraepithelial lesions and cervical cancer.

Year	Author	Population	Sample	CST	Metabolites
2020	(Borgogna et al., 2020)	HPV-negative participants (n = 13) and HPV-positive participants (n = 26)	Vaginal swabs	CST-I	Higher concentrations of histamine, 3-n-acetyl-LL-cysteine-S-yl acetaminophen, and gammaaminobutyrate
				CST-III	High levels of 3-n-acetyl-L-cysteine-S-yl acetaminophen
				CST-IV	Low levels of heme, glycerophosphorylcholine, and oxidized glutathione
2019	(Ilhan et al., 2019)	78 premenopausal, non-pregnant women and grouped as follows: healthy HPV-negative (n = 18) and HPV-positive participants (n = 11), low-grade squamous intraepithelial lesions (n = 12), high-grade squamous intraepithelial lesions (n = 27) and invasive cervical carcinoma (n = 10)	Cervicovaginal lavages and vaginal swabs	CST-IV	1) Higher levels of cadaverine, putrescine, tyramine, tryptamine, agmatine, and glutathione synthesis intermediate, 2-hydroxybutyrate, branched chain amino acid metabolism product, alpha-hydroxy-isovalerate, and L-isoleucine metabolism product, 2-hydroxy-3-methyl-valerate 2) Lower levels of nucleotides adenosine and cytosine and xenobiotics such as 2-keto-3-deoxy-gluconate and 1,2,3-benzenetriol
2019	(Kwon et al., 2019)	Normal women (n = 18), cervical intraepithelial neoplasia two or three patients (n = 17), and cervical cancer patients (n = 12)	Cervical swabs	CST-IV	1) Cervical cancer patients: enriched in peptidoglycan biosynthesis (ko00550) pathway 2) Cervical intraeptithelia neoplasia 2/3 patients: enriched in ko00300 (lysine biosynthesis), ko00680 (methane metabolism), and ko05211 (renal cell carcinoma)

CST, Community state types; HPV, human papillomavirus.

The interaction between the host and reproductive tract microorganisms forms a metabolic network that participates in the formation of the local tumor environment during the process of continuous HPV infection and cancer progression (Ilhan et al., 2019). In HSILs and CC, the vaginal microbial community disrupts amino acid and nucleotide metabolism in a manner similar to that in BV (Ilhan et al., 2019). Compared with healthy individuals, the abundance of lipid metabolites in the vaginas of ICC patients is higher (Ilhan et al., 2019; Szewczyk et al., 2019). This phenomenon may be related to the interaction between microbes and the host, which activates the carcinogenic pathways in the tumor microenvironment and increases cell proliferation and cell membrane synthesis, thus enhancing the carcinogenic activity of the microflora. Changes in the cervicovaginal microbial community cannot only change the cervicovaginal metabolome but also further affect immunity and participate in cancer progression (Ilhan et al., 2019). For example, glycochenodeoxycholate (GCDC) is a key metabolite of host-*Lactobacillus* cometabolism and can inhibit vaginal flora disorders (Ilhan et al., 2019). A decrease in GCDC and *Lactobacillus* species in CC patients leads to a decrease in the ability to induce inflammation and toxic reactions and further leads to a weakened antitumor effect.

The interaction between immunity and metabolites forms a special tumor microenvironment. In the CIN group, the concentrations of IL-8, IL-10, and nitric oxide in cervicovaginal secretions were higher than those in the control group, indicating that these mediators play a role in the tumor immune microenvironment (Tavares-Murta et al., 2008). Since IL-8 is a Th1-type cytokine and has a proinflammatory effect and IL-10 is a Th2-type cytokine and has an anti-inflammatory effect (Fernandes et al., 2015), the interaction mechanism between nitric oxide and the two needs to be further studied. ICC patients with high genital inflammation (high IL-1α, IL-1β, IL-8, MIP-1*β*, CCL20, regulation on activation normal T-cell expressed and secreted (RANTES), and TNFα expression) had the strongest correlation with lipids. An increase in plasmalogens and long chain polyunsaturated fatty acids in ICC not only indicates abnormal cell metabolism but also has a proinflammatory cytokine precursors effect, inducing abnormal gene expression and disordered cytokine production (Ilhan et al., 2019). Metabolites are also closely related to CC progression and tumor cell growth. CC is characterized by an immunosuppressive microenvironment and Th2-type cytokines (Bedoya et al., 2014). In females with CC, Th2-type cytokines (IL-10 and IL-13) induce the expression of arginase (ASE), which converts L-arginine into L-ornithine and polyamines, and a reduction in L-arginine is related to the downregulation of the immune response, further promoting tumor progression (Bedoya et al., 2014). Therefore, an increase in polyamines in the vagina flora of CST IV HPV-positive patients is a metabolic feature that HPV uses to escape host immunity and promote tumor progression (Borgogna et al., 2020). More research is needed to support the impact of HPV infection, cervical lesions, and the direct mechanism of action between the bacterial flora, metabolites and immunity in the cervicovaginal secretions of patients with CC on tumor progression and tumor metastasis.

### Endometrial Cancer

In 2018, there were an estimated 382,069 new cases and 89,929 deaths related to corpus uteri cancer (Bray et al., 2018). Endometrial cancer (EC) is a perimenopausal and postmenopausal tumor, divided into two categories: type I and type II (Troisi et al., 2018). Type I EC is the most common (Troisi et al., 2018). Environmental factors, including obesity, inflammation, postmenopausal estrogen metabolism imbalance and estrogen therapy, are the main risk factors for the development of type I EC (Laniewski et al., 2020). Type II EC is rare and is mainly related to endometrial atrophy (Troisi et al., 2018). Environmental factors are related to changes in the intestinal and vaginal microbiomes. The close relationship between the flora, estrogen metabolism and obesity indicates the potential role of the microbiome in the etiology of EC (Laniewski et al., 2020). EC is also closely related to PID, and an imbalance in the vaginal flora can cause PID through ascending infection, so an imbalance in the vaginal flora may be indirectly related to EC (Ness et al., 2005; Yang et al., 2015; Champer et al., 2018).

The reproductive tract microbiota is involved in the pathogenesis of EC (Laniewski et al., 2020). In 2016, Walther-Antonio et al. (2016) analyzed the genital tract flora of 17 patients with EC, four with endometrial hyperplasia and 10 with benign uterine diseases and found that in the EC cohort, *Porphyromonas* sp. was common in the vagina and cervix, *Bacteroides* and *Faecalibacterium* sp. were common in the endometrium, and *Bacteroides* sp.was common in the ovary. The endometrial microbiota of the EC and hyperplasia cohorts was similar but differed to some degree from that of the benign cohort. The endometrial hyperplasia and benign cohorts had different microbiota structures, indicating that the microbiota plays a role in the early stages of cell transformation (Walther-Antonio et al., 2016). EC patients usually have a high vaginal pH. The detection of vaginal *Atopobium vaginae* and *Porphyromonas* sp. combined with a high vaginal pH is statistically correlated with EC (Walther-Antonio et al., 2016). Studies have found that *Atopobium vaginae* can induce proinflammatory cytokines and antimicrobial peptides, cause chronic inflammation and local immune disorders, promote *Porphyromonas* sp. infection in cells, destroy normal cell regulatory functions, and ultimately lead to carcinogenic processes (Walther-Antonio et al., 2016; Laniewski et al., 2020). The link between *Atopobium vaginae* and *Porphyromonas* sp. supports the link between BV-related bacteria, immunity and EC (Laniewski et al., 2020).

EC has a unique endometrial metabolic signature. In 2017, Altadill et al. (2017) studied the metabolomics of endometrial tissue samples from 39 EC patients and 17 healthy women and found lipids, kynurenine, endocannabinoids and RNA editing pathway disorders in EC patients. Through further research on RNA editing pathways, we found that adenosine deaminases acting on RNA2 (ADAR2) are overexpressed in EC and are positively correlated with tumor aggressiveness. ADAR2 may contribute to the carcinogenicity of EC and can be used as a potential marker for EC treatment. However, whether the genital tract flora metabolites involved in EC carcinogenesis remains to be studied. It is known that the concentration of hydroxybutyric acid in the reproductive tract of BV patients is elevated. In the intestine, SCFAs (such as hydroxybutyrate) induce Treg cells through histone deacetylases (HDACs) and exert an immunosuppressive effect in innate immune cells (McMillan et al., 2015; Chen and Stappenbeck, 2019). Whether hydroxybutyrate in the reproductive tract induces immune suppression through HDACs and promotes the growth of endometrial tumors requires further *in vitro* experiments.

### Ovarian Cancer

Ovarian cancer (OC) is one of the deadliest malignant tumors in women and the main cause of death from gynecological malignancies (Turkoglu et al., 2016; Laniewski et al., 2020). In 2018, an estimated 295,414 new cases of OC were diagnosed worldwide, and 184,799 women died from the disease, ranking fifth among cancer-related deaths (Bray et al., 2018). More than 80% of patients have advanced disease, and the five-year overall survival rate is between 15 and 45% (Turkoglu et al., 2016). Similar to EC, chronic infection of sexually transmitted pathogens and ascending infection of genital tract inflammation are related to the occurrence of OC (Shanmughapriya et al., 2012; Idahl et al., 2020).

In 2019, Nene et al. (2019) first published a study on the presence of abnormal uterine flora in women with OC or at risk of OC and found a strong correlation of OC or the *breast cancer susceptibility gene 1* (*BRCA1*) mutation status with participants aged <50 years and those with a non-*Lactobacillus* dominant microbiota. Women who have used oral contraceptive pills or combined hormones for more than 5 years are more likely to have *Lactobacillus* dominance and a lower risk of OC than women who are using oral contraceptive pills or women who have used combined hormones for less than 5 years. Compared with the healthy surrounding ovarian tissue of the same individual, OC tissue has unique microbial characteristics (Banerjee et al., 2017). Potentially pathogenic intracellular microorganisms, such as *Brucella* spp., *Chlamydia* spp. and *Mycoplasma* spp., are present in 60–76% of ovarian tumors (Banerjee et al., 2017). In addition, an increase in *Proteobacteria* and *Firmicutes* phyla in ovarian tumors, especially an increase in *Actinobacteria* phyla, may cause double-stranded breaks by releasing bacterial toxins (such as colibactin and cytolethal distending toxin) and directly damage cellular DNA (Banerjee et al., 2017; Nene et al., 2019). In addition, several pathogenic viruses, intracellular bacteria, fungi and parasites also exist in ovarian tissue (Banerjee et al., 2017). These microorganisms may induce cancer through direct or indirect mechanisms (Laniewski et al., 2020). It is known that the integration of the HPV genome into the human genome is an important reason for the development of CC. In 2017, Banerjee et al. (2017) found HPV signals in the tumor tissues of OC patients, and there was also an integration phenomenon. For example, HPV16 has the largest number of viral integration sites in human chromosomes. It can be integrated into various intronic regions and genetic regions within 56 kb upstream of many cancer-related human genes. In addition, the coding sequence of the E1 gene of HPV18 is integrated in the intronic region of the non-coding RNA gene of the host chromosomes. All of the above factors may lead to the dysregulation of gene expression and participate in the occurrence and development of cancer (Banerjee et al., 2017). This research provides new ideas for exploring the molecular mechanism of OC.

It is known that the metabolic characteristics of OC cells, OC tissues, and ascites are significantly changed and are closely related to tumor tissue energy utilization, reproductive tract inflammation, the invasion and migration of OC cells, and the chemotherapy resistance of OC (Fong et al., 2011; Poisson et al., 2015). However, there are still few studies on the correlation between the characteristics of microbial metabolism in the reproductive tract and OC. The ovary is the end organ of the upper genital tract and is affected by the ascending bacteria of the genital tract, the bacteria of the ovary and the flora in the abdominal cavity. Whether the metabolic characteristics of the above bacteria are related to the occurrence and development of ovarian tumors and the carcinogenicity of tumors, leading to a unique tumor microenvironment, needs further research. Metabolites are closely related to tumor immunity and tumor development (Turkoglu et al., 2016; Clifford et al., 2018; Szewczyk et al., 2019). Whether the metabolites of the genital tract flora of OC patients participate in tumor immunity and host antitumor immunity, which affects the occurrence, development and metastasis of OC, still needs further research.

## Conclusion

The host reproductive tract microenvironment includes microorganisms, metabolites and immunity, and the balance of the interactions among them is essential to maintain reproductive health. Existing research on the relationship between female reproductive tract microbes or immunity and reproductive tract inflammation, pregnancy, and tumors is becoming increasingly detailed. In contrast to research on intestinal metabolites, research on female reproductive tract metabolites is still in the preliminary stage. Moreover, the reproductive tract metabolic characteristics of AV, reproductive dysfunction, EC, and OC still need more research at present, as relevant data are lacking. Additionally, the relationship between microbial metabolites and host immunity in inflammation, pregnancy, and tumors of the female reproductive tract is relatively unclear, and it may become a new direction for future research. According to the current research, BV-related/CST IV bacteria and the microenvironment formed by the reproductive tract have the most comprehensive research on the adverse pregnancy outcomes and tumor pathogenesis. The reproductive tract microenvironment produced by BV/CST IV not only participates in the ascending infection causing PID, which leads to an increased risk of infertility, miscarriage, and premature delivery, but also participates in the increased risks of precancerous lesions and malignancy of the reproductive tract. The pathogenic mechanism of adverse pregnancy outcomes and tumor diseases in the AV microenvironment of the reproductive tract needs to be further explored. Molecular detection technology combined with immune and metabolomics can be used to better describe the function and metabolic status of the flora, infer the possible pathogenic pathways and immune response status, and analyze the complicated relationship of the local microenvironment with inflammation, pregnancy, and tumor diseases. This method is also the best way to study the pathogenic mechanism and disease characteristics of female reproductive tract inflammation, pregnancy, and tumor diseases in the reproductive tract microenvironment. The study of microorganisms, metabolites, and immunity in the microenvironment of the reproductive tract under different diseases (and then the development of targeted therapies for the above three) was the main purpose of this article. At present, there are many studies on the treatment of microorganisms with antibiotics and probiotics. Studies have proven that probiotic supplementation is very helpful in reducing the risk of inflammation, adverse pregnancy outcomes, and cancers (Nene et al., 2019; Laniewski et al., 2020) and improving the ability to respond to cancer treatments and quality of life (Postler and Ghosh, 2017; Champer et al., 2018; Laniewski et al., 2020). However, microbial therapy is still prone to relapse and associated with a high risk of recurrence. Whether targeted therapy for metabolites (Sévin et al., 2015) and immunity (Ventriglia et al., 2017; Deshmukh and Way, 2019) can be used to treat diseases or improve the effect of microbial therapy still needs further research. Therefore, studying the role and mechanism of reproductive tract flora, metabolites, and immunity in disease pathogenesis will aid in disease diagnosis and treatment and improve female reproductive health.

## Author Contributions

HL, CH, and FX conceived the study question, and all authors were involved in the study design. HL created the first draft of the manuscript. YZ, CW, HYL, and AF made substantial contributions to drafting the article and revising it critically. All authors contributed to the article and approved the submitted version.

## Funding

This work was supported by Tianjin Municipal Science and Technology Commission Special Foundation for Science and Technology Major Projects in Control and Prevention of Major Diseases (Grant No. 18ZXDBSY00200), General Project of the National Natural Science Foundation of China (Grant No. 82071674) and Tianjin Health Science and Technology Project (Grant No. KJ20003).

## Conflict of Interest

The authors declare that the research was conducted in the absence of any commercial or financial relationships that could be construed as a potential conflict of interest.
